# Risk factors for blood transfusion in patients undergoing hysterectomy for stage I endometrial cancer

**DOI:** 10.1007/s00423-025-03629-4

**Published:** 2025-02-17

**Authors:** Abdelrahman Yousif, Hatem S. Mohamed, Anna Woodham, Mohanad Elchouemi, IIana Chefetz

**Affiliations:** 1 Department of Obstetrics and Gynecology, Paul L. Foster School of Medicine, Texas Tech University Health Sciences Center El Paso, El Paso, TX 79905 USA; 2 Rollins School of Public Health, Emory University, Atlanta, GA 30322 USA; 3 Paul L. Foster School of Medicine, Texas Tech University Health Science Center, El Paso, TX 799905 USA; 4 Department of Biomedical Sciences, Mercer University School of Medicine, Macon, GA 31207 USA

**Keywords:** Endometrial cancer, Blood transfusion, Risk factors, Hysterectomy, Chemoresistance

## Abstract

**Purpose:**

To highlight the risk factors contributing to blood transfusion among patients undergoing surgical intervention for Stage I Endometrial Cancer (EC).

**Method:**

Using the American College of Surgeons National Surgical Quality Improvement Program database, a nationally validated database dedicated to improving surgical care, females over the age of 18 who underwent surgery for EC stage I between the years 2016–2022 were queried. The cohort was then characterized based on those who received blood transfusion 72 h postoperatively.

**Results:**

27,183 patients with endometrial cancer who received surgical management were identified. 668 (2.5%) of those patients received blood transfusions. A multivariate logistic model found that a medical factor low preoperative Hct % (aOR 22.4, 95% CI[17.7, 28.3]; *p* < 0.001) and surgical factors such as 180 min or more of operative time (aOR 3.38, 95% CI[2.77, 4.14]; *p* < 0.001), larger uteri of 250–500 g (aOR 1.93, 95% CI[1.48, 2.49]; *p* < 0.001) and ≥ 500 g (aOR 2.35, 95% CI[1.77, 3.12]; *p* < 0.001), and abdominal approach compared to laparoscopic (aOR 6.36,95% CI[4.95, 8.18]; *p* < 0.001) were significantly associated with receiving blood transfusion.

**Conclusion:**

Many significant risk factors were found to be associated with blood transfusions in patients with Stage I endometrial cancer. These findings allow surgeons to proactively prepare adequate measures for patients who may require blood transfusions when they undergo surgery for endometrial cancer.

## Introduction

Endometrial cancer (EC)is the most common gynecologic malignancy among women in the United States [1]. In a meta-analysis, it was found that 26.5% of endometrial cancer patients have anemia even before their surgical treatment [2]. Anemia of cancer (AOC) is considered an independent risk factor for perioperative complications among cancer patients. Perioperative blood transfusion among gynecologic malignancies patients ranges from 3–77% [3]. A study by Swift et al. on 61 531 gynecologic oncology cases found that ovarian cancer had the highest rate of transfusion at 62% followed by EC at 33% and cervical cancer respectively with 4% [4].

Moreover, it is shown that blood transfusion was associated with poorer surgical and oncologic outcomes. For example, blood transfusion was found to be associated with increased rates of infections and metastasis among colorectal cancer patients [5]. Among EC patients, patients who underwent transfusion had worse 5 year-survival compared to non-transfused patients in a study on 263 endometrial cancer patients above the age of 60 years old. More advanced FIGO stages were associated with a higher likelihood of blood transfusion compared to early-stage EC patients [6].

Several studies showed that blood transfusion is an independent risk factor for perioperative morbidity among gynecologic oncology patients including ovarian, uterine, and cervical cancer patients [6–8]. Our analysis focuses on stage I EC patients, as it accounts for the majority of EC cases and is mostly treated surgically, making it a large group to investigate transfusion-related outcomes. Surgical management via simple hysterectomy either through an open surgery or a minimally invasive approach is the mainstay treatment for this subset of patients.

Our study highlights the risk factors including demographics, perioperative characteristics, and medical conditions contributing to blood transfusion in this cohort. The National Surgical Improvement Quality Program (NSQIP) is a comprehensive multi-institutional database [9] that includes blood transfusion events up to 72 h postoperatively as well as perioperative information on surgical patients. The goal of this study was to identify risk factors that require transfusion following surgery among stage I EC patients.

## Methods

### Ethical considerations

Institutional review board approval is not required.

Our study is a retrospective database cohort study that the American College of Surgeons’ ACS NSQIP database. Participating sites follow strict guidelines to ensure data quality and anonymity of patient information.

The NSQIP 2016–2022 participant files were used to identify patients above 18 years old with stage I EC. We identified malignancy patients using the variable “gynecologic cancer case” yes or no, and EC patients were identified via the “Uterine corpus stage” variable.

Demographic data, medical comorbidities, and perioperative data collected included the following: age, Body Mass Index (BMI), tobacco use, American Society of Anesthesiologists classification (ASA), race, ethnicity, functional status, bleeding disorders, congestive heart failure (CHF), hypertension (HTN), diabetes mellitus (DM), dialysis-dependent renal failure, prior abdominal surgeries, prior pelvic surgeries, operative approach, operative time, preoperative hematocrit (HCT), and preoperative platelet count. Age was dichotomized into two groups with 60 years as the cutoff point reflecting the risk of EC. Functional status was classified as “dependent” if the health status was recorded as partially or totally dependent otherwise “independent”.

Our primary outcome was blood transfusion event that was defined as transfusion of packed Red Blood Cells (RBC)s in the first 72 h postoperatively.

Data were imported and analyzed using R Studio (R version 4.3.0; R Core Team (2023); Vienna, Austria). We performed descriptive statistics to describe the overall cohort by blood transfusion outcome. Then, we performed univariate analysis and multi-logistic regression to examine the association between clinical variables of interest and our outcome of blood transfusion. For categorical variables, we used chi-square test and independent t-test for continuous variables. Independent variables which were clinically significant or had a significance level of *P* ≤ 0.1 in the univariate analysis were included in the multivariable logistic regression model. Using the “glm” function we constructed the multivariable logistic model to predict the occurrence of a blood transfusion (dependent variable) with *P* values < 0.05 deemed as statistically significant.

## Results

Between 2016 and 2022 we identified 27,183 patients with Stage I EC who underwent surgical management. Six hundred sixty-eight (2.5%) had received blood transfusion. As shown in Table 1 the transfused EC patients in comparison to the non-transfused were slightly younger (61 years vs.63 years; *p* = 0.006), had a higher proportion of non-white race (44% vs. 31%; *p* < 0.001) and more Hispanic ethnicity (8.1% vs. 6%; *p* = 0.001). HTN, DM, and endometriosis were the top medical comorbidities among the whole cohort at 55.3%,23.4% and 5.6% respectively with higher proportions among the transfused group with for endometriosis (8.2% vs. 5.6%; *p* = 0.003).

Preoperative history showed that 60% of our cohort were ASA class 3–5, 30.1% had prior abdominal surgery, 47.5% pelvic history and 1.1% were functionally dependent with higher proportion among EC patients who received transfusion (3.5% vs. 1.0%; *p* < 0.001).

**Table 1:** Characteristics and Univariate Analysis of Endometrial Cancer patients by blood transfusion status

Characteristic	Blood Transfusion			*p*-value^2^
	Overall, *N* = 27,183^1^	No, *N* = 26,515 (98%)	Yes, *N* = 668 (2.5%)	
Age, Mean (SD)	63 (11.1)	63 (11.1)	61 (13.2)	**0.006**
Race, *n* (%)				**< 0.001**
White	18,640 (68.6%)	18,268 (68.9%)	372 (55.7%)	
Non-White	8,543 (31.4%)	8,247 (31.1%)	296 (44.3%)	
Hispanic ethnicity, *n* (%)				**0.001**
No	21,074 (77.5%)	20,595 (77.7%)	479 (71.7%)	
Yes	1,658 (6.1%)	1,604 (6.0%)	54 (8.1%)	
Unknown	4,451 (16.4%)	4,316 (16.3%)	135 (20.2%)	
Body Mass Index, *n* (%)				**< 0.001**
Underweight	136 (0.5%)	127 (0.5%)	9 (1.4%)	
Normal	3,242 (12.0%)	3,152 (11.9%)	90 (13.7%)	
Overweight	5,080 (18.8%)	4,919 (18.6%)	161 (24.4%)	
Obesity	18,614 (68.8%)	18,215 (69.0%)	399 (60.5%)	
Unknown	111	102	9	
Parity, *n* (%)				**0.017**
0	7,752 (28.5%)	7,550 (28.5%)	202 (30.2%)	
1	4,454 (16.4%)	4,349 (16.4%)	105 (15.7%)	
2	7,963 (29.3%)	7,788 (29.4%)	175 (26.2%)	
3	4,285 (15.8%)	4,189 (15.8%)	96 (14.4%)	
4	2,729 (10.0%)	2,639 (10.0%)	90 (13.5%)	
H/o Smoking, *n* (%)	2,080 (7.7%)	2,016 (7.6%)	64 (9.6%)	0.058
H/o Hypertension, *n* (%)	15,034 (55.3%)	14,663 (55.3%)	371 (55.5%)	> 0.9
H/o Diabetes Mellitus, *n* (%)				**0.003**
IDDM^3^	1,792 (6.6%)	1,727 (6.5%)	65 (9.7%)	
NIDDM^3^	4,559 (16.8%)	4,442 (16.8%)	117 (17.5%)	
H/o CHF, *n* (%)	259 (1.0%)	241 (0.9%)	18 (2.7%)	**< 0.001**
H/o Bleeding disorder, *n* (%)	381 (1.4%)	345 (1.3%)	36 (5.4%)	**< 0.001**
H/o COPD, *n* (%)	537 (2.0%)	519 (2.0%)	18 (2.7%)	0.2
H/o Dialysis, *n* (%)	70 (0.3%)	61 (0.2%)	9 (1.3%)	**< 0.001**
Steroid use, *n* (%)	524 (1.9%)	506 (1.9%)	18 (2.7%)	0.14
Functional status, *n* (%)				**< 0.001**
Dependent	292 (1.1%)	269 (1.0%)	23 (3.5%)	
Independent	26,850 (98.9%)	26,208 (99.0%)	642 (96.5%)	
Unknown	41	38	3	
ASA, *n* (%)				**< 0.001**
Class 1	430 (1.6%)	423 (1.6%)	7 (1.0%)	
Class 2	10,459 (38.5%)	10,282 (38.8%)	177 (26.5%)	
Class 3–5	16,275 (59.9%)	15,792 (59.6%)	483 (72.4%)	
Unknown	19	18	1	
H/o Endometriosis, *n* (%)	1,533 (5.6%)	1,478 (5.6%)	55 (8.2%)	**0.003**
H/o Prior abdominal surgery, *n* (%)	8,182 (30.1%)	7,974 (30.1%)	208 (31.1%)	0.6
H/o Prior perlvic surgery, *n* (%)	12,903 (47.5%)	12,611 (47.6%)	292 (43.7%)	**0.049**
Preoperative Hematocrit level, *n* (%)				**< 0.001**
≥ 30%	26,449 (97.3%)	26,028 (98.2%)	421 (63.0%)	
< 30%	734 (2.7%)	487 (1.8%)	247 (37.0%)	
Operation time (minutes), *n* (%)				**< 0.001**
Less than 180	20,243 (74.5%)	19,928 (75.2%)	315 (47.2%)	
180 or more	6,940 (25.5%)	6,587 (24.8%)	353 (52.8%)	
Uterine weight (g), *n* (%)				**< 0.001**
< 250 g	23,618 (86.9%)	23,233 (87.6%)	385 (57.6%)	
250–500 g	2,495 (9.2%)	2,361 (8.9%)	134 (20.1%)	
≥ 500 g	1,070 (3.9%)	921 (3.5%)	149 (22.3%)	
Surgical approach, *n* (%)				**< 0.001**
Laparoscopic	19,428 (71.5%)	19,254 (72.6%)	174 (26.0%)	
TAH^4^	3,352 (12.3%)	2,981 (11.2%)	371 (55.5%)	
Other	4,403 (16.2%)	4,280 (16.1%)	123 (18.4%)	
Lymphadenectomy, *n* (%)	11,889 (43.7%)	11,749 (44.3%)	140 (21.0%)	**< 0.001**

^1^Mean (SD); *n* (%)

^2^Wilcoxon rank sum test; Pearson’s Chi-squared test; Fisher’s exact test

^3^Insulin dependent diabetes mellitus; Non-insulin dependent diabetes mellitus

^4^Total Abdominal Hysterectomy

*Multivariate logistic regression*

As for the surgical characteristics, higher percentage among the transfused group (53%) had 180 min or more of reported operative time compared to the non-transfused group (25%, *P* < 0.001), found to have larger uteri (>/=250 g) (42% vs. 12%; *p* < 0.001) [10]. In addition, abdominal approach was five times more frequent amount the transfused group (55% vs. 11%; *p* < 0.001). We also found higher percentage of patients with blood transfusion (37% vs. 1.8%; *p* < 0.001) had low preoperative HCT level (< 30%).

In the univariate analysis (Table 1), demographic factors and medical comorbidities like bleeding disorders, congestive heart failure, insulin-dependent DM, endometriosis, renal dialysis, preoperative functional status, and low preoperative HCT level were significantly associated with requiring blood transfusion. In addition, Abdominal hysterectomy as a surgical approach compared to laparoscopy, larger uterine size, and operative time of more than 180 min were significantly associated with increased likelihood of requiring blood transfusion and were included in the multivariate analysis.

**Fig. 1:**
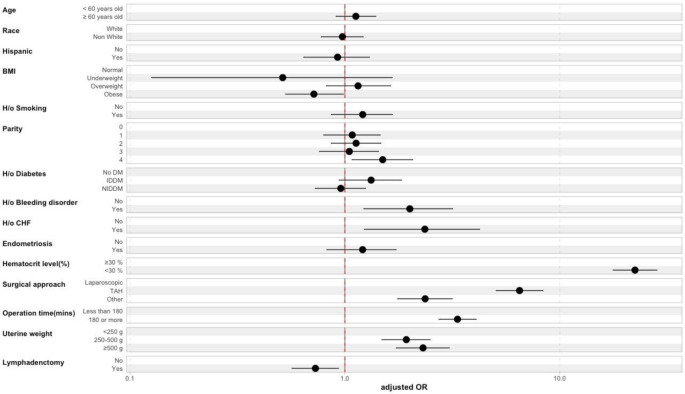
Plot of the Adjusted Odds Ratio and the 95% Confidence Interval for the dependent variable (Blood Transfusion) and the predictor variables

**Fig. 2:**
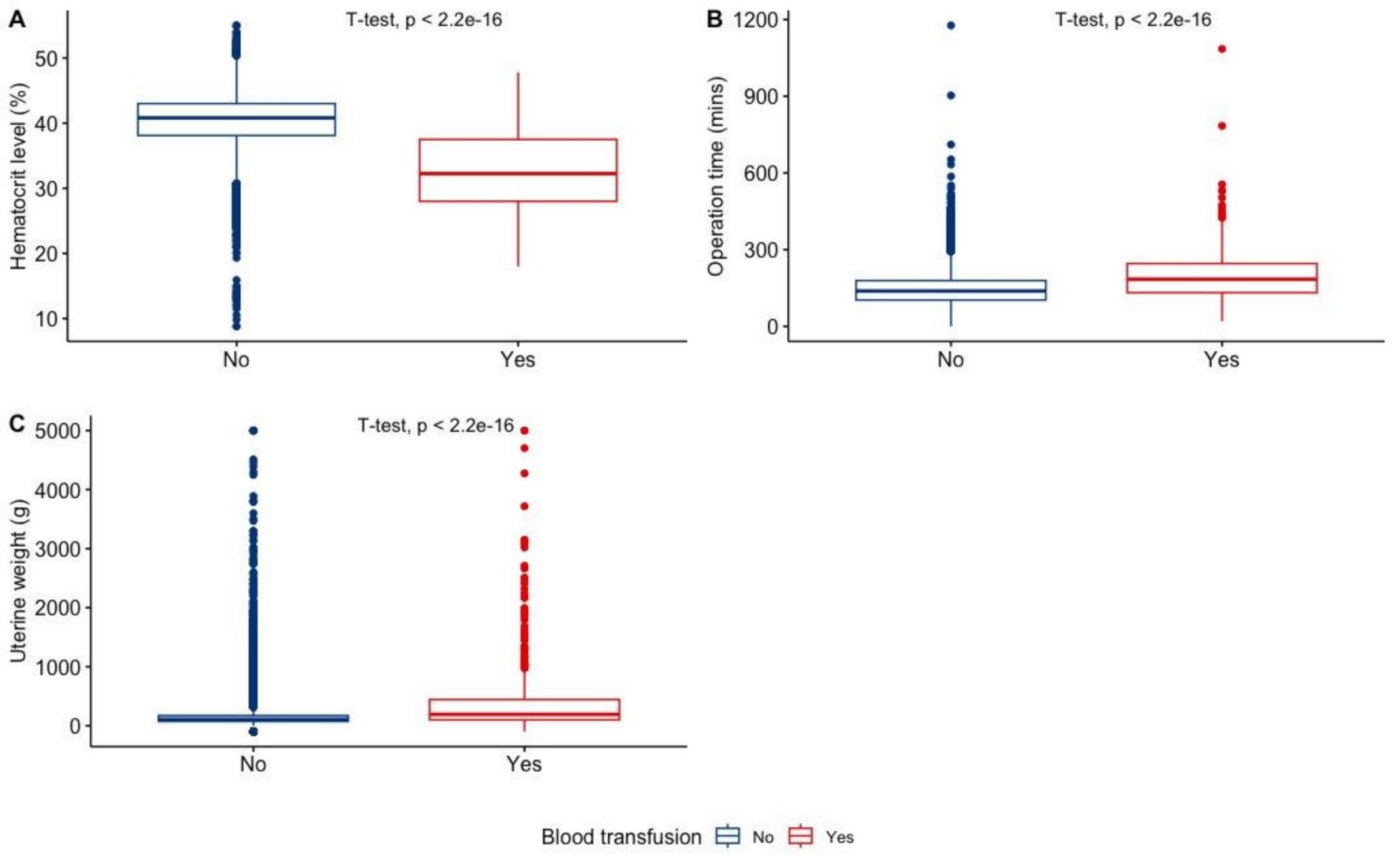
Boxplots of the (**A**) Hematocrit level (%); (**B**) Operation time (minutes); (C) Uterine weight (grams) as continuous variables between two groups of Endometrial cancer patients according to Blood transfusion Status. Difference in means calculated using student t’s test

After adjusting first for demographics, then medical and surgical characteristics, there was a significant association between blood transfusion and the following clinical variables (Table 2): history of bleeding disorders (aOR 2.01, 95% CI[1.23, 3.20]; *p* = 0.004), chronic heart failure (aOR 2.30, 95% CI[1.20, 4.15]; *p* = 0.008), dependent functional status (aOR 2.59, 95% CI[1.41, 4.47]; *p* = 0.001), and low preoperative HCT % (aOR 22.3, 95% CI[17.7, 28.2]; *p* < 0.001). In regard to surgical characteristics and operative time, 180 min or more of operative time (aOR 3.38, 95% CI[2.77, 4.14]; *p* < 0.001), larger uteri of 250–500 g (aOR 1.93, 95% CI[1.48, 2.49]; *p* < 0.001) and ≥ 500 g (aOR 2.35, 95% CI[1.77, 3.12]; *p* < 0.001), and abdominal approach compared to laparoscopic (aOR 6.36,95% CI[4.95, 8.18]; *p* < 0.001) were associated with an increased likelihood of blood transfusion in our cohort (Figs. 1 and 2).

**Table 2:** Multivariable Logistic Regression Analysis of Factors associated with blood transfusion. Significance level set at *P*-values ≤ 0.05

Variables	aOR^1^	95% CI^1^	*p*-value
Age			
< 60 years old	—	—	
≥ 60 years old	1.13	0.91, 1.39	0.3
Race			
White	—	—	
Non White	0.97	0.78, 1.22	0.8
Hispanic			
No	—	—	
Yes	0.93	0.64, 1.30	0.7
BMI			
Normal	—	—	
Underweight	0.51	0.13, 1.66	0.3
Overweight	1.15	0.82, 1.63	0.4
Obesity	0.72	0.53, 0.98	**0.036**
H/o Smoking			
No	—	—	
Yes	1.21	0.86, 1.66	0.3
Parity			
0	—	—	
1	1.08	0.80, 1.46	0.6
2	1.13	0.86, 1.47	0.4
3	1.05	0.76, 1.43	0.8
4	1.50	1.08, 2.07	**0.014**
H/o Diabetes			
No DM	—	—	
IDDM	1.33	0.94, 1.83	0.10
NIDDM	0.96	0.73, 1.25	0.8
H/o Bleeding disorder			
No	—	—	
Yes	2.01	1.23, 3.17	**0.004**
H/o CHF			
No	—	—	
Yes	2.36	1.23, 4.24	**0.006**
Endometriosis			
No	—	—	
Yes	1.21	0.82, 1.73	0.3
Hematocrit level(%)			
≥ 30%	—	—	
< 30%	22.4	17.7, 28.3	**< 0.001**
Surgical approach			
Laparoscopic	—	—	
TAH	6.50	5.07, 8.36	**< 0.001**
Other	2.36	1.76, 3.16	**< 0.001**
Operation time(mins)			
Less than 180	—	—	
180 or more	3.34	2.74, 4.09	**< 0.001**
Uterine weight			
< 250 g	—	—	
250–500 g	1.93	1.49, 2.50	**< 0.001**
≥ 500 g	2.31	1.74, 3.06	**< 0.001**
Lymphadenectomy			
No	—	—	
Yes	0.73	0.57, 0.93	**0.013**

^1^aOR =Adjusted Odds Ratio, CI = Confidence Interval

## Discussion

In a large cohort of stage I EC patients in the NSQIP database, we found that the overall incidence of blood transfusion is 2.5% in EC patients undergoing surgical management. This rate is comparable to Backes et al.‘s 3% transfusion rate in 503 EC patients undergoing robotic surgical staging but lower than the 7.5% observed by Uccella et al. in 358 EC patients [11, 12]. Additionally, perioperative transfusion rates across gynecological malignancies vary widely, ranging from 3 to 77%, with the upper range reflecting rates observed in ovarian cancer patients undergoing extensive cytoreductive surgeries [13–15].

Patients who had blood transfusion were found to have a medical history significant for bleeding disorder, CHF, and preoperative HCT < 30%. In addition, patients were more likely to undergo abdominal approach, have longer operative time (> 180 min), and larger uteri (> 250 g).

Optimization of preoperative medical conditions such as preoperative anemia via iron supplementation leading up to the surgery could lower the risk of blood transfusion among EC patients in a different study [16].

One systematic review found that about 25 to 75% of patients undergoing major oncologic surgery were found to have preoperative anemia [17]. Preoperative anemia contributes to the increased likelihood of blood transfusion among cancer patients. In our cohort preoperative hematocrit < 30 was significantly associated with blood transfusion. Furtherly, blood transfusion is shown to be associated with increased short- and long-term morbidity and mortality. For example, a study by Prescot et al. found increased postoperative complications following blood transfusions such as pneumonia, sepsis, and mortality among 8906 gynecologic cancer patients [7]. Similar results were obtained by Halabi et al. among colorectal cancer surgery patients [18]. A study by Anic et al. on 152 EC patients showed that blood transfusion was independently associated with decreased 5 years of progression-free survival (PFS) and overall survival rate (OS) [6]. This phenomenon can be explained by various reasons including oxidative stress, inflammation, and immunomodulatory effects. The presence of residual leucocytes, cytokines, and soluble mediators and changes in blood during storage can contribute to transfusion-related immunomodulation that suppresses response to cancer treatments [19]. Similarly, transfusion can trigger oxidative stress that fuels cancer growth and accordingly causes reduced PFS and OS. This effect can be diminished by ensuring anaerobic storage conditions, scavenging toxic compounds, and improving the antioxidant protection of the stored blood [20]. Finally, we hypothesize that transfusion can trigger the activation of Toll-like receptors (TLRs) and the innate immune system. Earlier studies showed TLR activation can trigger chemoresistance and enrichment of Cancer Stem Cells [21].

In addition, blood transfusion has inherent potential complications such as allergic reactions, fever, hemolytic reactions, and bloodborne infections [22].

Given the increased likelihood of blood transfusion and its associated perioperative morbidity and mortality, exploring different risk factors for blood transfusion and blood transfusion protocols has been an area of interest for cancer surgeons.

For instance, a study by Ackroyd et al. in ovarian cancer patients showed that a model including age, preoperative HCT, platelets count, abdominal approach, the presence of ascites and/or disseminated cancer, and potential advanced surgical procedures (colectomy or exenteration) with a score of </= 6 was associated with less than 17% risk of blood transfusion [23]. Similar findings citing age, preoperative hemoglobin, and laparotomy were observed among patients undergoing benign gynecologic surgeries [24, 25].

Another study by Swift et al. on patients with ovarian, uterine, and cervical cancers found that patients undergoing laparotomy approach for their surgeries are associated with an increased risk of blood transfusion compared to their counterparts undergoing minimally invasive approaches [4]. Similarly, our findings suggest that patients who underwent a total abdominal hysterectomy compared to laparoscopic approaches were five times more likely to receive blood transfusion.

Several studies compared liberal transfusion policies to restrictive policies among gynecologic and non-gynecologic patients. Liberal transfusion policy is defined as blood transfusion with Hb < 10 mg/dl and maintained at 10 to 12 mg/dl compared to Hb < 7 mg/dl and maintained at 7 to 9 mg/dl in the restrictive policy [26]. The findings of the *Transfusion Requirement in Critical Care* trial by Hébert et al. in 1999 were practice changing after showing restrictive blood transfusion policies were as effective as liberal transfusion protocols [27]. In a study by Boone et al. on 582 gynecologic oncology patients receiving 2276 transfusions under a restrictive blood transfusion policy, protocol-compliant and noncompliant patients showed similar rates of postoperative infections, thrombotic events, and 30-day mortality events [28]. Conflicting results showed that liberal policy was associated with fewer postoperative composite endpoints in surgical oncology patients meanwhile Boone et al. found that restrictive policies among the gynecologic oncology population were without worsening morbidity and mortality rates [28].

Larger uteri often necessitate more extensive dissection and prolonged operative time, both of which could exacerbate blood loss [29, 30]. The association between larger uterine weights and increased transfusion risk may partially stem from benign gynecological conditions such as uterine fibroids, which contribute to uterine enlargement and vascularity, thereby elevating the potential for intraoperative blood loss [30]. Similarly, endometriosis, another common benign condition, can increase surgical complexity through extensive adhesive disease. The need for more adhesiolysis in these cases often results in prolonged operative times and increased tissue trauma, both of which significantly contribute to higher transfusion rates [31]. These findings highlight the importance of preoperative planning and individualized surgical approaches in patients with such conditions to mitigate these risks.

However, a limitation of our study is the lack of detailed information on the presence or severity of these conditions in the database. Future research should seek to explore these associations further by integrating detailed clinical data on prevalent benign gynecological conditions, such as uterine fibroids and endometriosis, to better understand their contributions to surgical complexity, intraoperative blood loss, and transfusion risk.

Our study acknowledges several limitations. Primarily, it is constrained by the inherent characteristics of the extensive national population-based database utilized. Furthermore, there is minimal to no capacity to ascertain the clinical context surrounding the administration of blood transfusions in the treated individuals. With the involvement of over 90 institutions, there is potential for variability in blood transfusion protocols employed across different entities. The voluntary nature of participation in the database might introduce selection bias, and the patient demographic within the database could deviate from the broader population; however, the potential impact of this is likely diminished due to the substantial number of participating institutions and patient volume. While NSQIP data is rigorously collected by trained surgical clinical reviewers directly from medical records, it is not impervious to abstraction errors which could impose limitations on the study outcomes.

In conclusion, this study provides a comprehensive analysis of the risk factors associated with blood transfusion in patients undergoing surgery for Stage I endometrial cancer (EC), the most frequently diagnosed stage of this disease. Over the study period from 2016 to 2022, we identified a relatively low transfusion rate of 2.5%. However, key factors associated with an elevated transfusion risk included prolonged operative time, increased uterine weight, low preoperative hematocrit levels, a history of abdominal surgeries, bleeding disorders, and congestive heart failure. These findings underscore the complexity of perioperative management in this patient population.

The strong association between low preoperative hematocrit levels and transfusion risk highlights the critical need for future research on preoperative optimization strategies. Prospective studies exploring interventions such as iron supplementation, erythropoietin therapy, and nutritional optimization could provide valuable insights into reducing transfusion rates. Additionally, the role of advanced surgical techniques, such as robotic-assisted hysterectomy, warrants further investigation. Robotic surgery, with its precision and minimally invasive approach, holds promise for reducing blood loss and transfusion requirements, particularly in patients with larger uteri or surgically complex cases. Interventional trials examining blood conservation strategies, such as intraoperative cell salvage and restrictive transfusion protocols, could also refine perioperative care and improve outcomes.

Our findings further indicate that patients requiring transfusions often experience longer hospital stays, underscoring the broader implications of transfusion-related risks on healthcare resource utilization and patient recovery. By emphasizing the importance of preoperative optimization and individualized surgical planning, this study provides actionable insights to enhance patient safety, optimize resource allocation, and reduce healthcare costs.

As the prevalence of EC continues to rise, ongoing research into transfusion risk factors and mitigation strategies will be essential. A deeper understanding of these associations will enable clinicians to better predict and manage transfusion needs, ultimately improving perioperative outcomes and quality of care for patients with Stage I EC.

## Data Availability

Original data can be accessed at American College of Surgeons National Surgical Quality Improvement Program database.

## Abbreviations

EC: Endometrial cancer
MIS: Minimally invasive surgical
